# Bilateral frosted branch angiitis as the presenting sign of antiphospholipid antibody syndrome

**DOI:** 10.1186/s12348-016-0089-9

**Published:** 2016-06-10

**Authors:** Edward H. Wood, Robert W. Wong

**Affiliations:** Byers Eye Institute at Stanford, Stanford University School of Medicine, 2452 Watson Court, Palo Alto, CA 94303 USA; Austin Retina Associates, 801 West 38th Street, Austin, TX 78705 USA; Texas A&M Health Science Center, Bryan, TX USA

**Keywords:** Retinal vasculitis, Frosted branch angiitis, Antiphospholipid antibody syndrome, Hydroxychloroquine

## Abstract

**Background:**

“Frosted branch retinal angiitis” is an encompassing term for a rare, typically bilateral diffuse retinal periphlebitis that may occur in a number of varying conditions. To our knowledge, we report the first case of frosted branch angiitis as the presenting sign of antiphospholipid antibody syndrome in a 28-year-old woman.

**Findings:**

This study is a retrospective case report and literature review. Serial fundus photos, fluorescein angiogram, and ocular coherence tomography taken were before and after treatment, showing resolution of diffuse retinal perivascular sheathing and macular edema along with marked improvement in visual acuity 4 months after the treatment with corticosteroids.

**Conclusions:**

Frosted branch angiitis can be seen in association with antiphospholipid antibody syndrome. Prompt recognition and treatment with corticosteroids may result in good visual prognosis, and long-term immunosuppression and additional anticoagulation may be beneficial to prevent recurrence.

## Findings

### Introduction

“Frosted branch retinal angiitis” is an encompassing term for a rare, typically bilateral diffuse retinal periphlebitis that may occur in a number of varying conditions. To our knowledge, we report the first case of frosted branch angiitis in a patient with the antiphospholipid antibody syndrome.

### Case report

A 28-year-old Hispanic female with past medical history significant for migraine headaches and remote spontaneous abortion presented to an outside hospital with a 2-month history of fatigue and severe headache. Initial bloodwork revealed an elevated erythrocyte sedimentation rate (ESR) at 41 mm/h. Magnetic resonance imaging (MRI) of the brain, computed tomography angiography (CTA) of the head and neck, and chest radiograph were unremarkable. Lumbar puncture disclosed an elevated cerebrospinal fluid (CSF) white blood cell count (WBC) with normal CSF protein and glucose. The patient was admitted to the hospital and empirically started on intravenous acyclovir for suspected viral meningitis. Polymerase chain reaction (PCR) testing of the CSF for herpetic disease as well as CSF culture returned negative. The diagnosis was modified to aseptic meningitis, and acyclovir was discontinued after 3 days of therapy. The consulting neurologist started the patient on a low dose of oral Solu-Medrol at 4 mg daily, and she was discharged from the hospital. Over the next 7 days, her headache persisted. Upon developing visual symptoms including floaters in both eyes, light sensitivity, and loss of vision in the right eye, she returned to the hospital. At that point, an ophthalmologic examination was sought.

**Fig. 1:**
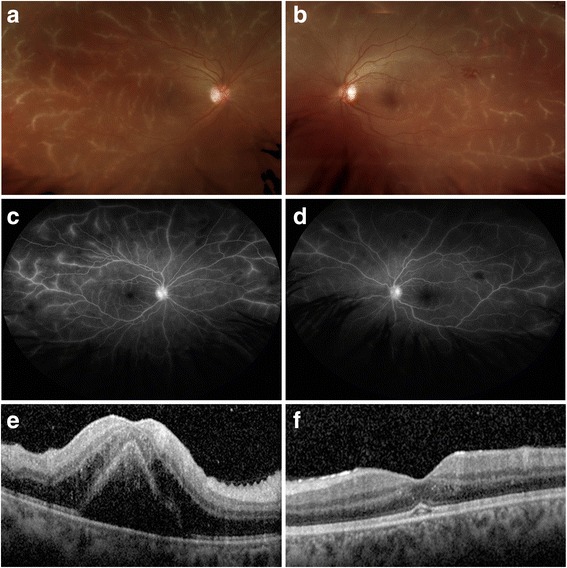
Fundus photography, fluorescein angiography, and optical coherence tomography of each eye at presentation. **a** Wide-field fundus photograph of the right eye (OD) showing diffuse retinal periphlebitis. **b** Wide-field fundus photograph of the left eye (OS) showing diffuse retinal periphlebitis. **c** Late-phase fluorescein angiogram of the right eye (OD) showing leakage of dye predominantly from the retinal veins and the optic nerve head without deceased transit time or evidence of vascular occlusion. **d** Late-phase fluorescein angiogram of the left eye (OS) showing leakage of dye predominantly from the retinal veins and the optic nerve head without deceased transit time or evidence of vascular occlusion. **e** Spectral domain ocular coherence tomography (SD-OCT) through the fovea of the right eye (OD) showing a large amount of cystoid macular edema and a serous foveal detachment. **f** Spectral domain ocular coherence tomography (SD-OCT) through the fovea of the left eye (OS) showing a smaller foveal detachment with trace intraretinal edema

On exam, the patient’s best-corrected visual acuity (BCVA) was count fingers on the right eye and 20/40 on the left. An afferent pupillary defect was noted on the right eye. On anterior segment examination, the patient demonstrated 1+ anterior chamber cell on the right with no anterior chamber cell on the left. She also had 2+ bilateral vitritis. Funduscopic exam revealed bilateral diffuse retinal periphlebitis resembling frosted branch angiitis (Fig. 1a, b). Fluorescein angiography (FA) showed slightly delayed retinal-artery-to-vein transit time of 10 s on the right eye. Late-phase FA demonstrated bilateral leakage of dye predominantly from the retinal veins and the optic nerve head (Fig. 1c, d). There was no evidence of capillary non-perfusion or neovascularization in either eye on FA. Spectral domain optical coherence tomography (SD-OCT) of the macula disclosed a large amount of cystoid macular edema and a serous foveal detachment on the right and a smaller foveal detachment with trace intraretinal edema on the left (Fig. 1e, f).

Subsequent workup for various infectious and inflammatory etiologies were significant for an elevated anti-nuclear antibody (ANA) titer at a 1:320 dilution with speckled homogenous pattern, an elevated anti-cardiolipin IgG at 85 GPL (with normal levels of anti-cardiolipin immunoglobulin m (IgM) and immunoglobulin A (IgA)), and both anti-beta 2 glycoprotein antibody and lupus anticoagulant being present. Other tests including C-reactive protein, human immunodeficiency virus (HIV) immunoassay, rapid plasma reagin, fluorescent treponemal antibody absorption, purified protein derivative (PPD), serum lysozyme, angiotensin-converting enzyme, human leukocyte antigen-B51, rheumatoid factor, anti-neutrophil cytoplasmic antibody, liver function tests, Sjogren’s syndrome antibody-A (anti-Ro/SSA), anti-La/SSB, anti-Smith, anti-ribonucleoprotein antibody (Anti-RNP), anti-centromere antibody, anti-topoisomerase I antibody (Anti-SCL 70), anti-double-stranded DNA antibody, complement C3 and C4, and aqueous humor PCR for herpes simplex virus, varicella zoster virus, cytomegalovirus, and *Toxoplasma gondii* were negative.

**Fig. 2:**
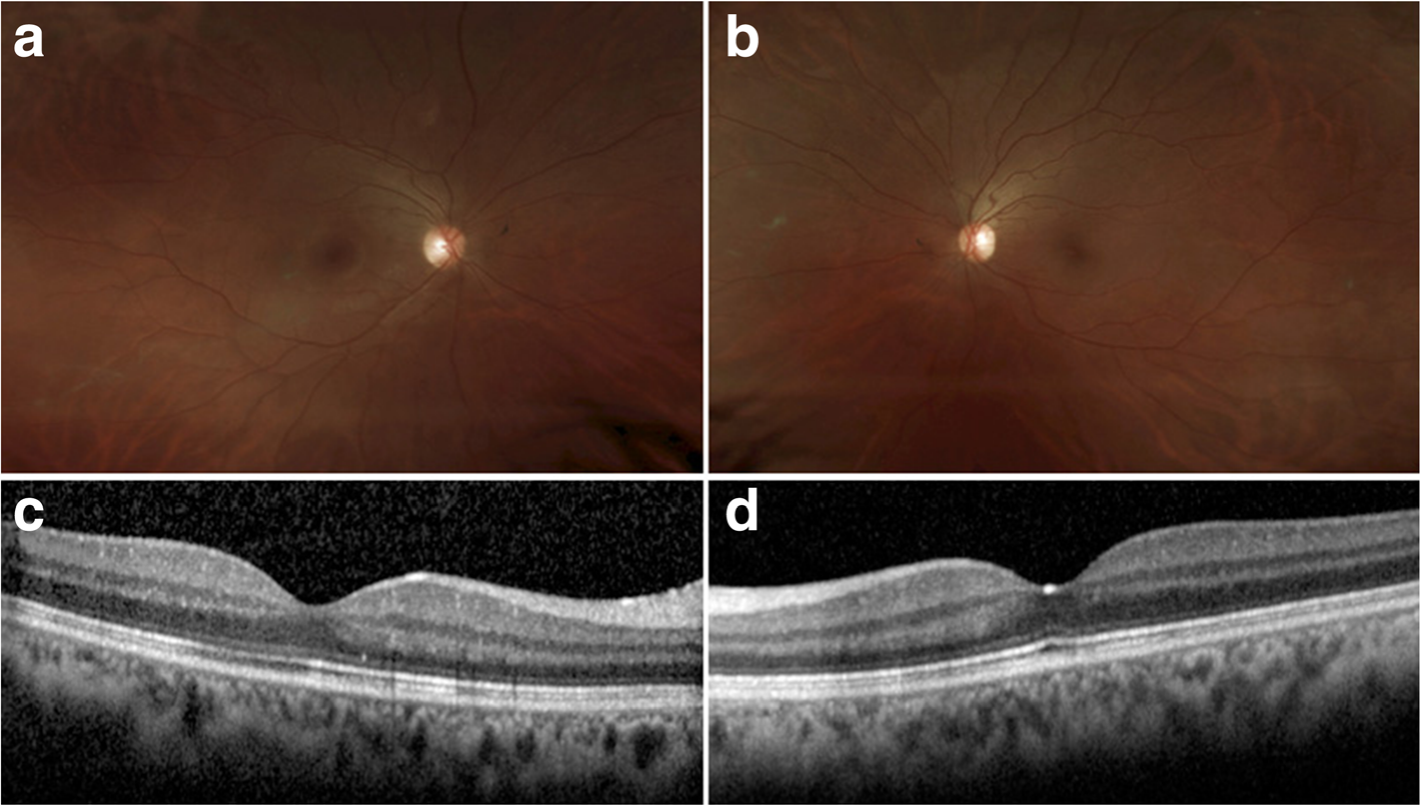
Fundus photography and OCT 4 months after treatment with corticosteroids. **a** Wide-field fundus photograph of the right eye (OD) showing markedly diminished perivascular sheathing. **b** Wide-field fundus photograph of the left eye (OS) showing markedly diminished perivascular sheathing. **c** Spectral domain ocular coherence tomography (SD-OCT) through the fovea of the right eye (OD) showing resolved macular edema. **d** Spectral domain ocular coherence tomography (SD-OCT) through the fovea of the left eye (OS) showing resolved macular edema

The patient was started on high-dose oral prednisone, at 1.5 mg/kg/day. One week after the initiation of treatment, the frosted branch angiitis improved. Four months after initiation of treatment, the patient’s VA was 20/20 in both eyes with resolution of the macular edema in both eyes (Fig. 2c, d) and markedly diminished perivascular sheathing bilaterally (Fig. 2a, b).

Rheumatology consultation was obtained, and it was found that the patient did not entirely meet full clinical diagnostic criteria for systemic lupus erythematosus (SLE). Subsequent hematology consultation disclosed that the patient met initial criteria for antiphospholipid antibody syndrome. Per recommendation by rheumatology, the patient was started on hydroxychloroquine initially at 400 mg daily as her systemic corticosteroids were tapered off.

Seven months after the initiation of treatment, the patient had persistently positive anti-cardiolipin IgG, anti-beta 2 glycoprotein antibody, and lupus anticoagulant. At this point, hematology confirmed the diagnosis of primary antiphospholipid antibody syndrome. Following a long discussion of the risks and benefits of long-term anticoagulation with the patient, she was started on Coumadin at 5 mg daily with regular international normalized ratio (INR) monitoring.

### Discussion

Ito et al. [1] first coined the term frosted branch angiitis in 1976 where they reported the appearance of thick perivascular sheathing in a 6-year-old boy’s fundus recalling imagery of frosted tree branches during an icy winter. Frosted branch angiitis may be idiopathic or associated with various systemic conditions including SLE [2, 3], Behcet’s disease [4], Crohn’s disease [5], cytomegalovirus (CMV) retinitis [6], herpes simplex type 2 infection [7], mycobacterium tuberculosis infection [8], *T. gondii* infection [9], various other viral and bacterial infections [10], aseptic meningitis [11], and blood dyscrasias such as leukemia [12] and lymphoma [13]. Additional retinal findings may include intraretinal hemorrhages, hard exudates, and serous exudative detachments of the macula and periphery. A proposed mechanism of retinopathy involves immune complex deposition in retinal vessels causing vasculitis [14]. Patients may complain of sudden onset of blurred vision, central scotomas, floaters, and photopsias, and most patients respond to systemic corticosteroid therapy with good recovery of visual acuity.

Antiphospholipid antibody syndrome (APS) is an acquired thrombophilic disorder in which autoantibodies are produced against a variety of phospholipids and phospholipid-binding proteins. Diagnostic criteria for APS include one clinical event and one laboratory finding. Clinically, a suspected patient must have suffered either vascular thrombosis (which may be venous, arterial, or small vessel) without significant evidence of inflammation in the vessel wall or have suffered pregnancy morbidity (fetal death after 10 weeks gestation, premature birth before 34 weeks associated with eclampsia, pre-eclampsia, or placental insufficiency, or three or more spontaneous abortions before the tenth week of gestation). Regarding the laboratory finding, the patient must have elevated levels of one of the following autoantibodies in serum or plasma on two or more occasions measured at least 12 weeks apart: lupus anticoagulant (LAC), anti-cardiolipin of IgG and/or IgM isotype (aCL), and/or anti-β2 glycoprotein I (aβ_2_GPI) antibody assays. These autoantibodies, collectively referred to as antiphospholipid antibodies (aPL), may arise from multiple mechanisms, including molecular mimicry between human B_2_GPI (a plasma protein that binds to negatively charged phospholipids and regulates coagulation) and similar molecules on pathogenic bacteria, as a reaction against certain medications (phenytoin and hydralazine among many), and other theorized mechanisms [15]. Generally, aPL may activate platelets, endothelial cells, and monocytes, leading to the inhibition of the fibrinolytic system, activation of the coagulation cascade, and possibly activation of the complement system [16]. The end effect of these pathways may lead to the thrombosis or fetal demise that is seen clinically in APS. The ophthalmic manifestations of APS are typically the result of vessel thrombosis as well, with vaso-occlusive disease involving the choroidal and retinal vasculatures as the most common locations for ocular involvement. Other findings such as anterior segment inflammation, episcleritis, scleritis, keratoconjunctivitis sicca, cranial nerve palsies, and ischemic infarcts of the visual pathway have been reported but are relatively uncommon [17].

In order to be diagnosed with definite SLE, the American College of Rheumatology has recommended that at least four of the 11 diagnostic criteria must be met. These criteria include (1) malar rash; (2) discoid rash; (3) photosensitivity; (4) oral or nose ulcers that are usually painless; (5) non-erosive arthritis in two or more joints; (6) cardiopulmonary involvement such as pericarditis or pleuritis; (7) neurologic disease including seizures or psychosis; (8) renal disease which may manifest as proteinuria or urine casts; (9) hematologic disorders including hemolytic anemia, leukopenia, lymphopenia, or thrombocytopenia; (10) immunologic disorder showing the presence of antibodies such as Anti-DNA, Anti-SM, and/or antiphospholipid antibodies (lupus anticoagulant (LAC), anti-cardiolipin of IgG and/or IgM isotype (aCL), and/or aβ_2_GPI); and (11) having a positive anti-nuclear antibody test (ANA) [18]. Following consultation with rheumatology, our patient demonstrated three clinical criteria and thus did not meet the full diagnostic criteria for SLE.

While retinal vasculitis is well observed in SLE, frosted branch angiitis has been described in only two patients with SLE [2, 3]. Both patients had previously met full diagnostic criteria for SLE without APS and presented with markedly decreased vision associated with prominent frosted branch periphlebitis. One patient received 1 g of IV methylprednisolone per day for 4 days, 2 units of packed erythrocytes, and 6 months of oral steroids and achieved marked visual improvement from HM OU to 20/60 OU. The other was treated with 500 mg of IV methylprednisolone daily for 3 days, followed by 3 weeks of 75 mg of prednisone along with 100 mg of azathioprine (patient’s chronic medication) daily, achieving nearly total remission of retinal lesions in 3 weeks with return of vision from 5/400 OU to 20/20 OU.

There exists a relationship between SLE and APS and clinically, patients may fall along a spectrum of disease. Patients with APS may have SLE. However, if patients with APS do not meet the American College of Rheumatology criteria for SLE diagnosis, they are diagnosed as having primary APS, as was the case in our patient. Gomez-Puerta et al. [19] found that that after median disease duration of 8.2 years, only 8 % of patients with primary APS developed SLE, with Coombs positivity conferring a statistically significant risk for the subsequent development of SLE. Montehermoso et al. [20] further solidified the relationship between SLE retinal vasculitis and APS, showing that 77 % of patients with SLE and retinal involvement had positive aPL titers, while only 29 % of SLE patients without retinal disease had positive titers. Stratified further, aPL antibodies are strongly associated with vaso-occlusive disease in SLE, a condition known as Hughes’ syndrome, and CNS manifestations of SLE [21]. Such patients should be initiated on lifelong anticoagulation therapy in concordance with APS management.

While SLE retinal vasculitis appears to be associated with the presence of aPL, frosted branch angiitis is very rare in the setting of aPL, and has not been previously described in APS. Abu El-Asrar et al. [22] reported two cases of retinal periphlebitis resembling frosted branch angiitis associated with non-perfused CRVO and positive aPL, but they did not meet the diagnostic criteria for APS. Despite systemic corticosteroid therapy, 80 mg/day of aspirin, and full panretinal photocoagulation, both patients developed rubeosis iridis and profound visual loss associated with neovascular glaucoma in one patient. Our patient, in contrast, had better visual and anatomical outcomes presumably due to the fact that we were able to identify and treat the retinopathy prior to the onset of widespread retinal ischemia and neovascularization.

The recommended primary treatment for APS is prevention of recurrent thrombosis or fetal morbidity with anticoagulation, and a discussion between the patient and physician should revolve around this decision. Once a patient has a proven thrombosis associated with aPL, lifelong anticoagulation is advisable. Many alternatives to Coumadin now exist, and the exact choice of anticoagulation should be tailored to the patient.

Hydroxychloroquine (HCQ) has also been shown to be protective against thrombosis in aPL-positive patients [23], and as in our patient, it may be reasonable to recommend HCQ for thrombosis prevention in APS as an add-on or alternative treatment option in cases resistant to other treatment modalities. While the drug is desirable for its steroid-sparing properties, a treatment period of roughly 6 months is required to reach steady state levels [24], and thus systemic immunosuppression with corticosteroids is often used in the acute setting.

In summary, to our knowledge, this is the first case of frosted branch angiitis seen in association with antiphospholipid antibody syndrome. With prompt treatment with corticosteroids, visual prognosis can be good. In these cases, long-term immunosuppression and additional anticoagulation may be beneficial to prevent recurrence. Appropriate consultations to hematology and rheumatology services are recommended. Ophthalmologists, rheumatologists, and hematologists should be aware of this possible association.

## Abbreviations

aCL, anti-cardiolipin; ANA, anti-nuclear antibody; Anti-La/SSB, Sjogren’s syndrome antibody-B; Anti-RNP, anti-ribonucleoprotein antibody; Anti-Ro/SSA, Sjogren’s syndrome antibody-A; Anti-SCL 70, anti-topoisomerase I antibody; APS, antiphospholipid antibody syndrome; aβ_2_GPI, anti-β2 glycoprotein I antibody; aPL, antiphospholipid antibodies (these autoantibodies are collectively referred to as aPL); BCVA, best-corrected visual acuity; CMV, cytomegalovirus; CSF, cerebrospinal fluid; CTA, computed tomography angiography; ESR, erythrocyte sedimentation rate; FA, fluorescein angiography; GPL, measurement unit; 1 g of IgG antibody; HCQ, hydroxychloroquine; HIV, human immunodeficiency virus; IgG, immunoglobulin g; IgM, immunoglobulin m; IgA, immunoglobulin A; INR, international normalized ratio; LAC, lupus anticoagulant; MRI, magnetic resonance imaging; PCR, polymerase chain reaction; PPD, purified protein derivative; SD-OCT, spectral domain optical coherence tomography; SLE, systemic lupus erythematosus; WBC, white blood cell
